# Point-of-care and self-testing for potassium: recent advances

**DOI:** 10.1039/d2sd00062h

**Published:** 2022-06-06

**Authors:** Tanya Hutter, Thomas S. Collings, Gratsiela Kostova, Fiona E. Karet Frankl

**Affiliations:** a Materials Science and Engineering Program & Texas Materials Institute, The University of Texas at Austin USA tanya.hutter@utexas.edu; b Kalium Health Ltd UK; c Cambridge Institute for Medical Research, University of Cambridge UK

## Abstract

Potassium is an important bodily electrolyte which is kept within tight limits in health. Many medical conditions as well as commonly-used drugs either raise or lower blood potassium levels, which can be dangerous or even fatal. For at-risk patients, frequent monitoring of potassium can improve safety and lifestyle, but conventional venous blood draws are inconvenient, don't provide a timely result and may be inaccurate. This review summarises current solutions and recent developments in point-of-care and self-testing potassium measurement technologies, which include devices for measurement of potassium in venous blood, devices for home blood collection and remote measurement, devices for rapid home measurement of potassium, wearable sensors for potassium in interstitial fluid, in sweat, in urine, as well as non-invasive potassium detection. We discuss the practical and clinical applicability of these technologies and provide future outlooks.

## Abstract 2

The review summarizes recent advances in technologies and devices for point-of-care and self-testing of potassium in venous blood, fingerprick blood, interstitial fluid, sweat, urine, and indirectly using ECG.

## Graphical abstract

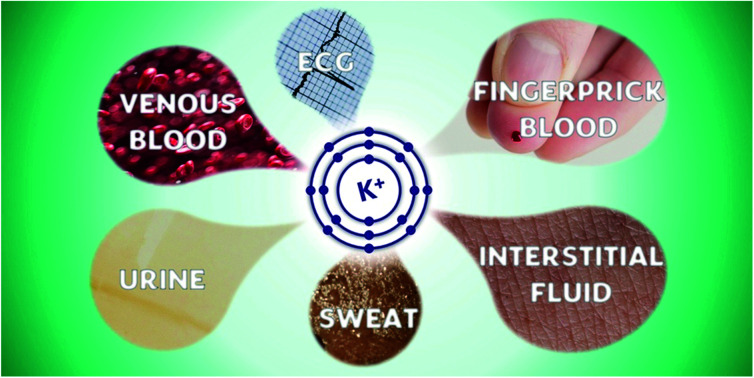

## 1. Introduction

For health, the human body depends upon the correct balance of intra- and extracellular electrolyte levels. Important examples include sodium, potassium, calcium, magnesium, phosphate and chloride. The reference range for blood potassium is 3.5–5.0 mmol L^−1^ (mM) (US: mEq L^−1^) and even small deviations are of particular importance as they can cause illness and even death.

Hypokalaemia and hyperkalemia are medical disorders in which the potassium level in the blood is lowered or raised, respectively. Both affect heart rhythm, and both may be life-threatening. Hypokalaemia causes disabling cramp, muscle weakness, paralysis, and/or seizures. As well as being a common effect of diuretics, hypokalaemia is found in a number of rare inherited disorders with varying prevalence of 1 : 40 000 to 1 : 1 000 000.^1^

Hyperkalaemia is much more dangerous, because the associated potentially fatal cardiac arrhythmia is usually asymptomatic. Hyperkalaemia is most frequently caused by impaired kidney function and/or antihypertensive medications used for the treatment of chronic kidney disease (CKD), heart failure or hypertension. Advanced CKD is estimated to affect more than 6% of adults,^2–4^ with hyperkalemia commonest in later stages. Heart failure and hypertension affect nearly half the adult population with an estimated 500 million people on antihypertensives,^5^ many of which carry the incumbent risk of hyperkalemia.

Due to the urgency of detection and treatment of severe hypokalaemia and hyperkalemia, frequent monitoring of blood potassium concentration is required, but often not achieved. These conditions are encountered both in hospital and in the community, but the currently approved *in vitro* diagnostic methods are restricted to professional use only. This has been largely due to technological limitations meaning that only a venous blood draw produces sufficient blood volume for measurement. However, within most current primary care practice settings there is an inevitable delay between venous blood draw and testing of the blood sample in a laboratory. This misses any opportunity for urgent medical intervention, with doctors only receiving results later that day or that week, and patients often never seeing the results. Furthermore, delay and transportation of the sample can lead to pseudohyperkalaemia, where *ex vivo* physiologic changes, and/or haemolysis, alter the balance between intra- and extracellular blood potassium.^6^ Therefore, a convenient point-of-care solution in primary care would be clinically very beneficial.

Providing patients with the means to measure their blood potassium concentration at any time, with sufficient accuracy to support clinical decision-making, could reduce the need for costly and inconvenient in-person test appointments. In line with existing clinical guidance^7,8^ it could also make it quicker and easier to adjust medications to optimise dose and cost at an individual level. Furthermore, having access to rapid blood potassium testing could support more effective use of emerging rapid potassium-lowering medications including patiromer (Vifor Veltassa)^9,10^ and sodium zirconium cyclosilicate (AstraZeneca Lokelma).^11,12^

Due to this apparent clinical need and the associated, potentially large, market opportunity, various academic groups and commercial companies have explored minimally invasive solutions to measure blood potassium either at the point-of-care, at home or on the body; although (and as a result) a programme of clinician and patient education would also be necessary to enable effective potassium monitoring outside of a clinic. This review outlines sensing technologies and devices known to be previously or currently under development and discusses their usability for clinical management and for home/self-monitoring.

## 2. Devices and technologies

### 2.1. Electrolyte detection technologies

**Fig. 1:**
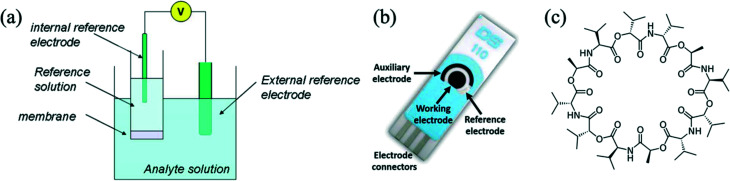
(a) An electrochemical cell for a potentiometric measurement with an ISE.^24^ (b) Screen-printed electrode from Metrohm DropSens.^25^ (c) Molecular structure of valinomycin, an ionophore that has high sensitivity and selectivity for potassium ions.

Ion-selective electrodes (ISE) are very useful electrochemical sensors for ions,^13–16^ as they are very sensitive,^17^ selective and are commercially available, allowing researchers to measure various ion concentrations down to as low as 10^−7^ mol L^−1^ (M). They are already integrated into laboratory instruments for blood and soil testing. The most basic ISEs consist of two electrodes connected to an electrical device that can measure potential difference (in volts) between the two electrodes. One electrode is termed a ‘reference electrode’, and the other is the ‘working electrode’. The working electrode is typically placed behind a polymer membrane containing an ionophore – a molecule that binds with a specified ion. The membrane is typically formed by mixing polymer, ionophore and a plasticizer. Traditionally, ISEs were liquid based, with an internal electrolyte reference solution as shown in Fig. 1a. Newer ISEs are solid-state and can therefore be miniaturized. Electrochemical screen-printed electrodes normally include the working, reference and auxiliary/counter electrodes on a polymeric or ceramic substrate (Fig. 1b). Those electrodes can be made ISEs *via* functionalisation of the working electrode with potassium selective chemistry. A common potassium ionophore is valinomycin, whose molecular structure is shown in Fig. 1c. Highly sensitive potassium sensors have been reported, capable of detecting concentrations as low as 10 μmol L^−1^ (ref. 18) and even down to 0.53 nmol L^−1^.^19^

In addition to potentiometric measurement, amperometric measurements can also be used to determine electrolyte concentrations, where the measurement involves a three-electrode configuration and the measurement of a current between the electrodes.^20^ Similarly, detection methods based on electrical impedance^21^ and optical response^22,23^ have been reported in the scientific literature. Those methods reply on a similar principle, where the ion of interest selectively binds to a molecule/ionophore, thus changing the electrical and/or optical response/color.

### 2.2. Point-of-care devices for measurement of potassium in venous blood

Devices intended for the point-of-care measurement of potassium are designed for use by professionals and rely on a venous blood sample to run the test. This sample type is the same as is used in long-established laboratory techniques and hence supports a relatively straightforward regulatory pathway and widespread clinical acceptance. Most point-of-care devices are sized for benchtop use and require a typical minimum blood volume of 100–200 microlitres (μL). Measurement is performed using ion-selective electrodes over the course of a few minutes, using a reusable cartridge with internal calibration. These devices are designed for low to moderate test throughput and the relatively high cost of the device and each cartridge are justified by the multiplicity of tests it can carry out.

**Fig. 2:**
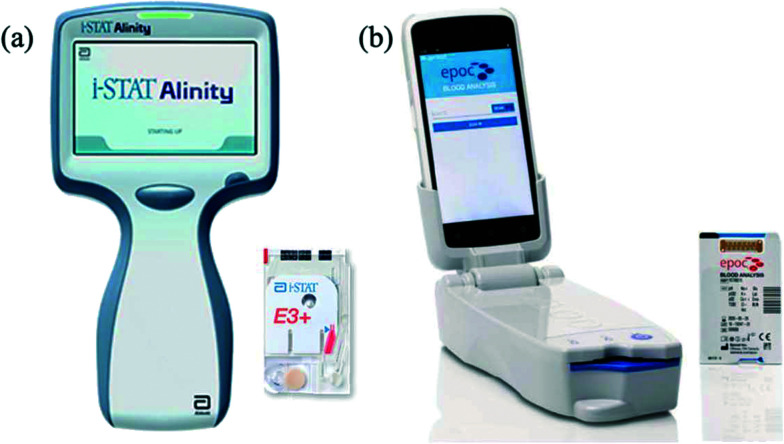
Portable point-of-care devices that can measure potassium in blood and approved for professional use only: (a) the Abbott i-STAT Alinity blood analyzer^27^ and i-STAT test cartridge which requires 65 μL of blood. (b) Epoc analyzer^28^ and Epoc disposable test cartridge, which requires 92 μL of blood.

Portable point-of-care devices exist, but require a minimum blood volume of over 60–90 μL, including Abbott i-STAT^26,27^ (Fig. 2a) and Siemens Epoc^28^ (Fig. 2b). These are approved by the US Food and Drug Administration (FDA) for professional use with capillary blood samples but are not approved for home/self-monitoring by patients. These devices have some practical limitations. The i-STAT cartridges require refrigerated transportation and storage; whereas the Epoc cartridges must be kept between 15–30 °C and have a shelf life of only five months from the time of manufacture. Moreover, the cost of the instrument and the single-use cartridges is high, which is not practical for regular home/self-testing by patients. The cartridges require a relatively large volume of blood, which cannot be obtained from a fingerprick sample without significant haemolysis.

It is important to note that obtaining a large drop of blood from a fingerprick, even with automated assistance, is highly problematic for potassium measurement – any squeezing of the finger to produce a large enough blood volume will result in rupture of red cells (haemolysis), thus artificially increasing the potassium concentration in the drop of blood. This is because extracellular potassium levels (∼4 mM) are much lower than those inside the red blood cells (∼140 mM). Therefore, if red blood cells rupture, they release potassium into the extracellular environment. Haemolysis is also a major concern for measurement of potassium in whole blood when sending blood samples to a lab for analysis; factors such as temperature, storage time and even handling, can cause haemolysis and falsely high potassium readings.^29–33^ Thus, the relatively large volume of blood required by current devices means that a fingerprick sample is inevitably haemolysed and therefore unsuitable for potassium measurement.

### 2.3. Devices for home blood collection and remote measurement

In the US, private home phlebotomy services are available whereby blood is collected *via* venous draw by a professional. In this case, conventional point-of-care or laboratory instruments are used to analyse the sample. In recent years, as especially during the ongoing coronavirus disease (COVID-19) pandemic, the popularity of home testing has increased, and many companies now offer remote blood collection, allowing blood samples to be obtained in patients' homes, eliminating the need for clinic or lab visits for a blood draw. Normally this includes a fingerprick blood collection kit, where the collected blood sample is then mailed to a lab for analysis. Such tests allow for a variety of different analytes and biomarkers to be measured, such as hemoglobin A1C (HbA1C) (diabetes), thyroid stimulating hormone (TSH) (thyroid health), cholesterol, vitamin D, and many more. The results are normally available 1–5 days later. However, as described above, the volumes required, and risk of haemolysis, always exclude potassium as an analyte.

Additional issues for potassium measurement are firstly that once removed from the body, blood cells continue to be physiologically active while glucose is still present, and so initially, potassium is pumped from the plasma into the cells and plasma levels actually fall. Once the energy source is exhausted, potassium begins to leak out of cells and into the plasma, so levels artefactually rise. This is a particular concern for patients living a long way from the point of analysis. Secondly, blood that is allowed to clot will appear to contain more potassium in the serum because during clotting, platelet activation releases potassium.

Currently, no home fingerprick collection potassium tests are available. Although potassium is one of the biomarkers offered by the Kitby Vitall kidney function home test,^34^ it requires a clinic visit (at additional cost) to obtain the blood. General Prognostics (USA)^35^ announced that it is in the early stages of development of a home test for blood potassium concentration, apparently based on collecting a dried spot of capillary blood which is then mailed to a laboratory for testing. However, no information is available about the launch date, how haemolysis or artefactual rises in potassium levels are avoided during clotting of the blood sample, or what instruments will be used to carry out analysis of the dried sample.

### 2.4. Devices for rapid home measurement of potassium

As outlined, blood collection at home followed by remote analysis does not support frequent monitoring of dynamic blood potassium levels. As a consequence, a number of companies have announced that they are developing solutions for rapid measurement and monitoring of blood potassium levels at home. All these are based on fingerprick capillary blood sampling, which is a widely accepted method for other rapid, frequent home tests such as blood glucometers.

PTS Diagnostics (USA)^36^ has a granted patent for ‘systems and methods for point-of-care detection of potassium’ (WO2020077344A1). This describes an enzymatic reaction with potassium ions to produce a mediator which is measured electrochemically to determine the concentration of potassium, a method that could be compatible with a conventional test strip and reader format. However, while the company markets a range of point-of-care diagnostic tests worldwide, no information has been published about a potassium test.

Jana Care (USA)^37^ has published papers describing a method of measuring potassium concentration using ion-selective optodes.^23^ These follow the established chemistry of ion-selective electrodes; however a colorimetric rather than electrical response is measured. The company currently markets point-of-care diagnostic tests for HbA1c and other blood markers, but a potassium test has not been announced despite announcing in 2021 a collaboration with AstraZeneca focused on home potassium monitoring.^38^

Kalium Health (UK)^39^ is developing a potassium test based on a low-cost, single-use test strip that is operated by a handheld electronic monitor. They have a patent for a particular electrochemical technique to improve sensor response time and performance (GB2582582A). The monitor will link to a smart app and digital data platform to provide patients with insights about their health and also transmit results to a care provider. The company announced in November 2021 that it had advanced into clinical testing to validate the core sensing technology. No launch date has been announced.

CardioRenal (France)^40^ is developing a product called ‘Tenor’ that consists of a home-based instrument that is linked to an app and a cloud system. The company had previously publicised the development of ‘Soprano’, a combined test for potassium, creatinine and hemoglobin. They have one patent related to sensing (FR3103279A1), which describes a manufacturing process for an ion-selective membrane. No launch date for either product has been announced.

Renalyse, formerly known as CreatSens (Spain)^41^ is developing a device to measure creatinine and potassium at home. The stage of the product development is unknown.

Lastly, an academic group at Graz University of Technology, Austria, in collaboration with Infineon Technologies Austria AG, published a paper in 2018 (ref. 42) describing an electrochemical sensor for potassium that also measures haemolysis as a quality control measure. No announcement has been made that the technology will be commercialised.

### 2.5. Potassium-sensing technologies for urine

In health, the kidneys are responsible for ensuring that extracellular levels of potassium are maintained within tight limits, and therefore widely varying amounts of potassium will appear in the urine, reflecting the combination of intake, various hormones such as aldosterone, and medications.^43^

When blood levels of potassium are found to be abnormal, reflecting either underlying health conditions such as kidney and cardiovascular disease and diabetes^44^ or medications used to treat not only these but other illnesses like cancer,^45^ measurement of potassium levels in urine becomes important in diagnosis, but is not a marker for blood levels. The usual method of evaluating urinary potassium levels is a 24-hour urine collection.^46^ However, this is often burdensome for patients and requires laboratory testing. A less accurate but easier test takes a ‘spot’ urine sample and compares potassium and creatinine concentrations. If this could be achieved at the point-of-care it would benefit both healthcare providers and patients in remote locations.

**Fig. 3:**
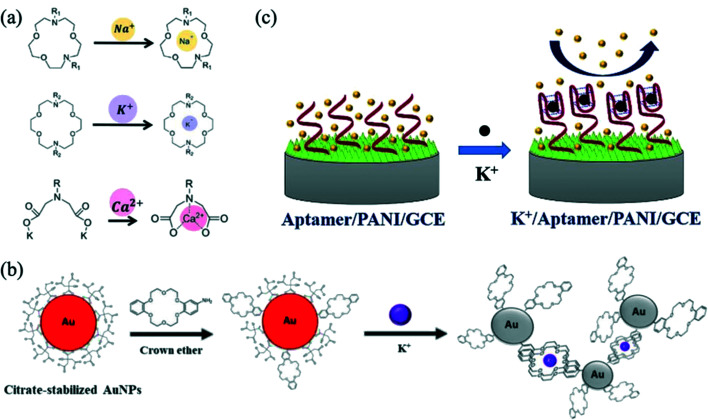
(a) Depiction of chelation mechanism of fluorescent probes for sodium, potassium and calcium ions. Reprinted with permission from ref. 47; Copyright 2020 Springer Nature Ltd: *Scientific Reports*. (b) Schematic illustration of the sensing mechanism of a paper-based colorimetric array test strip for the detection of potassium. Reprinted with permission from ref. 48; Copyright 2021 IOP Publishing Ltd. (c) Aptasensor mechanism where the electrochemical impedance changes when the aptamer–potassium complex is formed. Reprinted with permission from ref. 51; Copyright 2022 Elsevier.

Ghaderinezhad *et al.* (2020) created a paper-based fluorescent and colorimetric smartphone-enabled device based on trapping ions of interest within fluorescent crown ether probes (Fig. 3a).^47^ Their device was optimised to detect potassium as well as calcium, chloride, and nitrate. It was tested with artificial urine and a calibration curve for potassium in the range 0–250 mM was prepared from 6 repeats. Chitbankluai *et al.* (2021) also used a crown ether on modified gold particles to produce a colorimetric array paper test strip to quantify potassium ion levels in urine (Fig. 3b).^48^ This sensor was reported to work across a range of 0.0005–1 mM *via* naked eye visualisation. The use of ISEs is also a popular choice in the detection of potassium in urine. Kucherenko *et al.* (2020) created solid-state ISE sensors to detect urinary potassium and ammonium ions over the range 0.3–150 mM.^49^ The ISE was used to assess the dehydrative effects of exercise in a 22 year old and a 73 year old individual. Spot urine potassium levels rose in both. Dębosz *et al.* (2021) also used ISEs to determine the concentration of potassium, sodium and calcium synthetic samples in clinically relevant concentration range, again demonstrating applicability for measurement of urine samples.^50^ In contrast, Salehan *et al.* (2022) created an aptasensor which determined potassium levels in fluids successfully using electrochemical impedance spectroscopy.^51^ There, a polyanaline coating on a glassy carbon electrode was modified with a potassium selective aptameter. The single-stranded DNA aptameter folded into a G-quadraplex configuration in the presence of potassium ions (Fig. 3c). An increase in potassium concentration led to an increase in charge transfer resistance monitored using impedance spectroscopy. The concentration of potassium in three different urine samples was assessed with high accuracy.

These promising scientific results demonstrate the capability of various sensing technologies to accurately detect potassium (and other ions) in urine. However, assessment of urine potassium can only ever be an adjunct to blood measurements in assessing disease or medication effects, enabling clinicians to determine the role, or otherwise, of the kidneys or specific medications in a particular patient. In addition, potassium is lost from the body in stool and sweat, and abnormalities in these functions will also be reflected in how much potassium appears in the urine. Thus, urine potassium concentration cannot be extrapolated to calculate blood values.

### 2.6. Wearable sensors for interstitial fluid potassium measurement

Interstitial fluid (ISF) fills the spaces between cells, and it exchanges nutrients and waste with blood contained in microscopic capillaries. ISF is vital to nutrient exchange between cells, preserving homeostasis of the cellular microenvironment, and plays a central role in the immune system. Currently, ISF is not used routinely in clinical settings despite its rich biomolecular content and important role in regulatory functions.

Microneedle technology uses very small needles or probes that access interstitial fluid without irritating deeper layers of the skin associated with pain, blood flow, or sensation. This allows minimally invasive measurement of biomarkers. Continuous glucose monitoring (CGM) is the most widely used implantable probe technology (Freestyle Libre 3, Abbott Laboratories and G7, Dexcom), allowing people with diabetes to measure their glucose levels continuously for up to two weeks and providing data to enable automated insulin administration (Omnipod 5, Insulet).

**Fig. 4:**
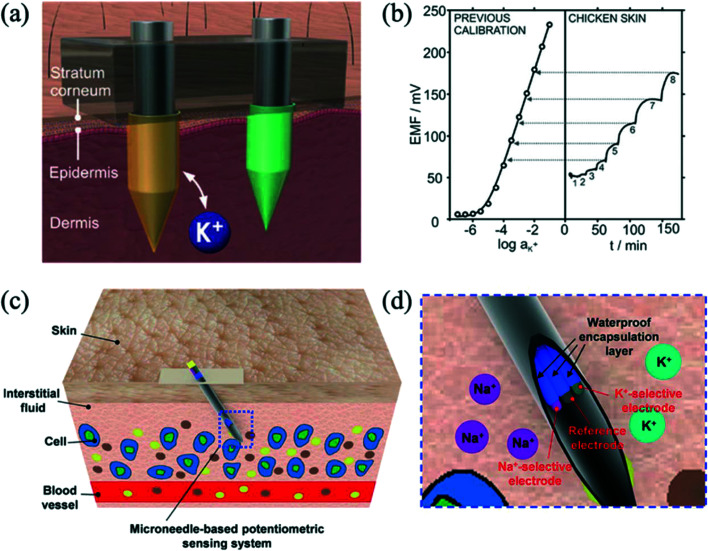
(a) Illustration of a microneedle patch inserted into the skin, allowing for potassium detection in the interstitial fluid within the dermis. Reprinted with permission from ref. 54; Copyright 2019 American Chemical Society. (b) Correspondence of the potassium concentrations (1–8 mM) measured during *ex vivo* experiments in chicken skin, with a previous calibration graph. Reprinted with permission from ref. 54; Copyright 2019 American Chemical Society. (c) Schematic illustration of the insertion of a microneedle-based potentiometric sensor into the skin. Reprinted with permission from ref. 55; Copyright 2021 American Chemical Society. (d) Illustration of a microneedle-based potentiometric sensing system consisting of a sodium ion-selective electrode, a potassium ion-selective electrode, and an Ag/AgCl reference electrode. Reprinted with permission from ref. 55; Copyright 2021 American Chemical Society.

Microneedles have been integrated with ion-selective electrodes to detect ions such as sodium and potassium, as well as other biomarkers, for measurement in dermal interstitial fluid.^52^ Miller *et al.* (2014) were the first to demonstrate a microneedle ISE sensor.^53^ They developed an ion-selective transdermal microneedle sensor for potassium ions, where a hollow microneedle with a microfluidic chip was used to extract interstitial fluid through a channel towards a downstream solid-state ISE. The device was tested using solutions with physiological potassium concentrations. Parrilla *et al.* (2019) reported all-solid-state platform for intradermal potentiometric detection of potassium in interstitial fluid.^54^ Solid microneedles were modified with different coatings and polymeric membranes were used to prepare both the potassium-selective electrode and the reference electrode. An illustration of a microneedle patch with the two electrodes inserted into the skin is shown in Fig. 4a. Sensor calibration and *ex vivo* measurement in chicken skin as a function of time with 1–8 mM concentrations is shown in Fig. 4b. Performance of the patch was tested *ex vivo* with chicken and porcine skin, and *in vitro* cytotoxicity experiments showed that the patch could be used for at least 24 hours without any adverse effect on skin cells. Li *et al.* (2021) demonstrated a stainless-steel hollow microneedle-based potentiometric sensing system for continuous monitoring of multiple electrolytes in skin interstitial fluids.^55^ These authors demonstrated monitoring of potassium and sodium ions by testing on phantom gel and chicken skin. Fig. 4c illustrates the insertion of the microneedle-based potentiometric sensor into the skin. Fig. 4d shows the sensing system, consisting of a sodium ion-selective electrode, a potassium ion-selective electrode, and an Ag/AgCl reference electrode.

Proton Intelligence (Canada)^56^ is developing a real-time wearable device to measure potassium levels in interstitial fluid. The company and the product are at an early stage of development.

Although those wearable sensor devices are very promising, microneedle-based potassium sensing has not been demonstrated *in vivo*, and potassium concentrations have not been measured directly in interstitial fluid. To the best of our knowledge there are no reported studies describing the concentration of potassium in human dermal ISF. We believe this is because obtaining such measurements is very difficult for several reasons, chief among which there are no existing devices that can provide an accurate measurement of potassium concentration in a microliter sample volume.

Therefore, before such technology can be used for any clinical applications, it is important first to correlate blood potassium levels with interstitial potassium levels and determine any time lag time between them. Exemplar studies of this sort include Gilanyi *et al.* (1988)^57^ who used corneal interstitial fluid samples and concluded that the Donnan equilibrium led to cation levels higher and anion values lower in interstitial fluid than in plasma. Also, potassium microneedle-based sensors need to be validated *in vivo* for monitoring dermal interstitial fluid in human subjects, which has not yet been done. If this type of methodology can be established, there will be several important benefits including no haemolysis affecting potassium reading, a pain-free experience and the possibility of continuous monitoring.

In addition to microneedles, there are other ways of obtaining ISF. Reverse iontophoresis involves the application of electrical potential or current between two electrodes on the skin which causes electro-osmotic flow of ISF from the inside of the skin onto the surface.^58,59^ Using this ISF extraction method and porcine skin, potassium was measured off-line using flame photometry.^60^ Also, ISF extraction can be done using sonophoresis, which employs an ultrasound to increase the permittivity of the skin to the ISF,^61^ and magnetohydrodynamics which induces ISF flow *via* external magnetic and electric fields.^62^ Those methods are still in early stages and have been mostly demonstrated for glucose measurement. Commercially, Kiffik Biomedical (US)^63^ is developing a method of obtaining continuous access to ISF, which works by forming stable microopenings through the epidermis. Once the microopenings have been created, the device uses negative pressure to extract interstitial fluid at a low flow rate. Those advances in obtaining dermal ISF will create opportunities for integration with potassium sensors in the future.

### 2.7. Wearable sensors for potassium in sweat

The popularity of wearable sensors for sports performance monitoring and in biomedical diagnostics is increasing, and so is the amount of research that has been conducted on developing potassium sensors as part of a wearable device. Sweat is an attractive medium because it is easily accessible and can provide non-invasive continuous monitoring. Chemical analysis of sweat could add useful physiological information to the information provided by currently available wearable sensors that measure heart rate, temperature and movement.

**Fig. 5:**
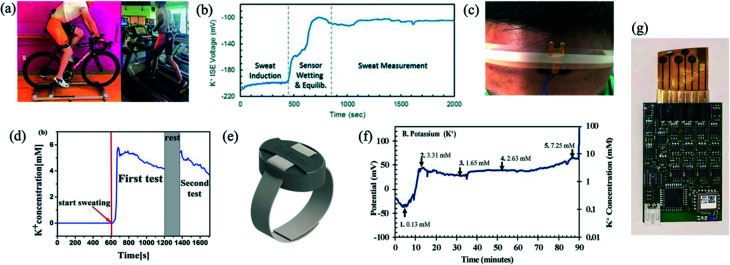
(a) Photographs of subject wearing an integrated sweat sensor during biking and running. Reprinted with permission from ref. 65; Copyright 2018 the Royal Society of Chemistry. (b) Integrated sweat potassium patch outputs during biking. Reprinted with permission from ref. 65; Copyright 2018 the Royal Society of Chemistry. (c) Photo of a sensor attached to the forehead. Reprinted with permission from ref. 66; John Wiley & Sons permission conveyed through Copyright Clearance Center, Inc. (d) Continuous measurement of potassium in sweat. Reprinted with permission from ref. 66; John Wiley & Sons permission conveyed through Copyright Clearance Center, Inc. (e) Fully enclosed 3D printed SwEatch platform. Reprinted with permission from ref. 68; Copyright 2020 Elsevier. (f) The SwEatch platform output for potassium for the duration of the 90 min on-body trial. Reprinted with permission from ref. 68; Copyright 2020 Elsevier. (g) Flexible wearable multi-electrode system for monitoring in compounds in sweat interfaced with read-out electronics. Reprinted with permission from ref. 69; Copyright 2021 Elsevier.

Ion-selective electrode sensors are often the primary method of detecting potassium in sweat. In general, most wearable sensors aim to achieve flexibility of the skin-patch, low data noise levels during performance, robustness and long-term stability, while being comfortable to wear on the skin. Gao *et al.* (2016) developed a completely integrated, mechanically flexible sensor array for sweat analysis, which measures glucose, lactate, sodium and potassium ions.^64^ Skin temperature was measured to adjust sensor responses. Alizadeh *et al.* (2018) also produced a sodium and potassium patch which can be used during exercise.^65^ The device transmitted signals wirelessly whilst maintaining low noise levels even during strenuous exercise such as running on a treadmill. Performance was demonstrated at a field test in a military training setting of 3-hours' duration. The ISE voltages of sodium and potassium sensors were recorded continuously; however, these were not translated to actual concentrations of the electrolytes. Fig. 5a shows photographs of a subject wearing an integrated sweat sensor during biking and running and Fig. 5b shows the output of the integrated sweat sensor as a function of time during biking. Liu *et al.* (2020) improved on this by developing a wearable detection platform based on high-electron-mobility transistors for monitoring of potassium and pH.^66^ Real-time monitoring of subjects who exercised for 33 minutes was performed. A photo of the sensor attached to a forehead is shown in Fig. 4c, and the continuous potassium measurement is plotted in Fig. 5d. At the onset of sweating, a rapid increase in concentration was observed, indicating the arrival of initial sweat. After a few minutes, the signal gradually diminished. During this time, potassium concentration decreased from about 5.5 to 4 mM. Coppedè *et al.* (2020) developed textile organic electrochemical transistors, which were functionalised with ion-selective coatings for potassium and calcium.^67^ The flexibility of the fabric was maintained after the coating, which is favourable for a sensor placed on human skin. However human sweat measurements were not conducted in this work. Pirovano *et al.* (2020) reported the development of the SwEatch platform, a wearable device for monitoring of both sodium and potassium simultaneously in human sweat.^68^ The device incorporates a microfluidic unit creating a sweat reservoir leading to the two sodium and potassium selective electrodes. Fig. 5e shows the fully enclosed 3D printed SwEatch platform, which was used to monitor athletes during a 90 min cycling exercise. During this time, sodium increased from 1.89 to 2.97 mM and potassium from 3.31 to 7.25 mM as shown in Fig. 5f, however authors state that validating data accuracy would still need to be performed in future. Criscuolo *et al.* (2021) also measured sodium and potassium simultaneously in sweat of five volunteers in a 30 minute interval using flexible electrochemical ISE based multi-sensing platform.^69^ Their *in situ* results agreed well with *in vitro* results, validating their sensors. They also ventured to detect lithium and lead in artificial sweat for diagnostics of other conditions. Their flexible wearable multi-electrode system for monitoring in compounds in sweat was interfaced with read-out electronics and is shown in Fig. 5g.

It is unfortunate that the variability of sweat electrolytes makes sweat unsuitable as a biomarker of blood levels. Vairo *et al.* (2017) showed that electrolyte concentration in sweat sampled following physical activity did not reflect concentrations in plasma.^70^ This is not to underestimate sweat's utility – for example, electrolyte composition of sweat is known to be abnormal in cystic fibrosis – sodium and chloride concentrations are much higher^71,72^ and sweat chloride concentration is a common diagnostic tool for cystic fibrosis.^73^ In health, electrolytes such as sodium and potassium could be related to hydration status, and characterization of sweat electrolytes of athletes could suggest modifications of diet and training habits.^74^

### 2.8. Non-invasive and indirect potassium detection

Non-invasive methods are used for pulse oximeters^75^ and some glucometers^76^ and use light or electrical signals to measure through skin. A challenge with developing a similar concept for non-invasive measurement for potassium is that potassium is a very small ion and does not have any unique spectral features that can allow optical measurement through skin.

An indirect estimation of potassium levels can be achieved by placing electrodes on the skin and measuring an electrocardiogram (ECG) signal over a short period of time.^77,78^ Such an approach was trialled by AliveCor,^79^ USA, a manufacturer of portable ECG monitors, who collaborated with Mayo Clinic on a retrospective study applying AI techniques to ECG traces to screen for hyperkalaemia.^80^ The company does not currently market a test for hyperkalaemia although it announced a research collaboration with AstraZeneca in 2021.^81^ Research in this area is ongoing; scientists are developing various algorithms in order to estimate blood potassium levels from ECG signals.^77,82,83^ To date, the accuracy needed for clinical and diagnostic purposes has not been demonstrated in significant numbers of human subjects using this method.

## 3. Discussion and future outlook

Clinical decisions regarding potassium levels are based on collecting whole blood samples and measuring plasma or serum concentrations. New technologies do offer the potential for measuring potassium in non-blood media such as sweat and interstitial fluid. However, the concentration of potassium in sweat does not correspond to blood, and cannot be used for medical/diagnostic purposes; interstitial fluid could provide a good medium for minimally invasive potassium measurement, but as yet there are no studies that demonstrate correlations between potassium concentrations in blood and interstitial fluid. Moreover, any time delay between changes in blood levels and a change in interstitial fluid would need to be accurately determined; a long delay would create a missed opportunity for intervention and would put the patient at risk. Potassium in urine is not directly related to blood levels, and of course patients with severe kidney disease may have ceased making urine altogether. Also, before any non-blood samples could be used for potassium determination for clinical decision-making, large clinical studies will have to be conducted to determine the suitability of that medium for practical/clinical usability.

Many research papers have been published in the scientific literature describing different variations of electrochemical and optical detection methods for potassium. The reported studies in the scientific literature are summarized in Table 1 with respect to the target fluid for which potassium sensors were developed, the fluid that was used for sensor testing, the device materials, methods, performance and the stage of development. However, most studies do not determine sensors' performance for potassium measurement capability in the relevant biological fluids – most are done with aqueous solutions that contain small amount of potential interferants or biomolecules that could affect the performance. For example, none of the sensors that were developed for *in vivo* potassium measurement in the interstitial fluid, were actually tested *in vivo*. It is therefore difficult to determine how such sensors might perform when tested with real fluids, and therefore their applicability for practical medical applications. More work needs to be done to validate these technologies in the relevant fluids for which they are developed.

**Table 1:** Summary table of the reported potassium sensor devices, target fluid for which they were developed, the fluid that was used for sensor testing, the device materials, methods, performance and the stage of development

Author (year)	Target fluid	Fluid analysed	Device materials (method)	Detection limit, concentration range, sensitivity and selectivity	Stage of development
Ghaderinezhad *et al.* (2020)^47^	Urine	Artificial urine	Crown ether on paper (colorimetry)	Calculated limit of detection of 2.39 mM	App for smartphone designed
Chitbankluai *et al.* (2021)^48^	Urine	Synthetic solutions	Crown ether modified gold nanoparticles on paper (colorimetry, visual inspection)	Testing range 0.005–1 mM	Sensor evaluated
Kucherenko *et al.* (2020)^49^	Urine	Human urine	Ion-selective membrane on graphene electrode (potentiometry)	Testing range 0.3–150 mM; sensitivity 53 mV per decade; selectivity coefficients reported	Long term storage assessed
Dębosz *et al.* (2021)^50^	Urine	Synthetic solutions and certified reference materials for urine	Ion-selective (valinomycin) octadecylamine-functionalized multi-walled carbon nanotube electrodes (potentiometry)	Testing range 0.1–100 mM; sensitivity 59.5 mV per decade; selectivity coefficients reported	3D-printed flow potentiometric measurement cell was designed
Salehan *et al.* (2022)^51^	Urine, serum	Human urine and serum	Potassium-selective aptamer DNA G-quadruplex conformation and polyaniline on a glassy carbon electrode (electrochemical impedance spectroscopy)	Limit of detection 3.7 × 10^−9^ mM; linear range from 10 pM to 60 μM; selectivity over other ions reported	3 urine samples and 3 serum samples were analysed
Miller *et al.* (2014)^53^	Interstitial fluid	Synthetic solutions	3D porous carbon electrodes integrated into a microfluidic channel, ISE microneedle sensor (potentiometry)	Linear range 0.1–10 mM, detection limit of 0.0022 mM; spiking with Na^+^ reported	*Ex vivo* testing
Parrilla *et al.* (2019)^54^	Interstitial fluid	Chicken and porcine skin, processed	ISE on functionalized multiwalled carbon nanotubes on a carbon-modified microneedle (potentiometry)	Limit of detection of 0.013 mM, linear response range 0.63–63 mM; selectivity coefficients reported	*Ex vivo* testing; *in vitro* cytotoxicity tested (safe for 24 hours)
Li *et al.* (2021)^55^	Interstitial fluid	Skin-mimicking phantom gel and processed chicken skin	Sodium, potassium ISFs and Ag/AgCl reference electrode in a stainless-steel hollow microneedle (potentiometry)	Testing range 0–15 mM; negligible interference from other ions	*Ex vivo*, measured concentrations successfully on animal skin
Wascotte *et al.* (2007)^60^	Interstitial fluid	Porcine ear skin, processed	Reverse iontophoresis for fluid extraction (flame photometry)	Testing range 0–6 mM	*In vitro* on porcine skin
Gao *et al.* (2016)^64^	Sweat	Human sweat	Potassium ISE multiplexed with sensors for sodium, glucose, lactate detection (potentiometry)	Testing range 1–32 mM; sensitivity 61.3 mV per decade; negligible interference from other ions	Detection during exercise demonstrated
Alizadeh *et al.* (2018)^65^	Sweat	Human sweat	Potassium ISE multiplexed with sensors for sodium detection (potentiometry)	Testing range 0.1–100 mM; sensitivity: 53.9 mV per decade; no response to Na+ ions	Detection during exercise demonstrated
Liu *et al.* (2019)^66^	Sweat	Human sweat	AlGaN/GaN high-electron-mobility transistors with potassium ion-selective membrane (electrical current)	Testing range 1 μM to 100 mM; sensitivity: 4.94 μA per decade	Detection during exercise demonstrated
Coppedè *et al.* (2020)^67^	Sweat	Potassium solutions	Textile organic electrochemical transistors modified with ion selective membrane (change in potential, electrical current)	Testing range 0.01–1000 mM; sensitivity and selectivity factor determined	Testing in various concentration solutions
Pirovano *et al.* (2020)^68^	Sweat	Human sweat	Potassium ISE multiplexed with sensors for sodium detection (potentiometry)	Testing range 0.1–316 mM; sensitivities 45.7 and 54.3 mV per decade	Detection during exercise demonstrated
Criscuolo *et al.* (2021)^69^	Sweat	Human sweat	Potassium ISE on platinum nanoflowers multiplexed with sensors for Li^+^, Pb^2+^, Na^+^, K^+^ and temperature detection (potentiometry)	Limit of detection in artificial sweat 3 mM; sensitivity in artificial sweat: 55.1 mV per decade	Detection during exercise demonstrated
Yasin *et al.* (2017)^77^	Non-invasive	Blood and ECG	Commercially available electrodes (Kardia, AliveCor) (ECG)	Blood potassium levels detected to within 9% of the blood test result	ECG data and blood samples collected from 18 patients undergoing chronic haemodialysis
Corsi *et al.* (2022)^78^	Non-invasive	Blood and ECG	12-Lead Holter H12+ Mortara Instrument Inc. (ECG)	Testing range 2.5–7.5 mM	Study population of 45 haemodialysis and 12 long QT syndrome type 2 patients
Palmieri *et al.* (2021)^82^	Non-invasive	Blood and ECG	12-Lead ECG Holter recording, H12+, Mortara Instruments (ECG)	Not reported	Study population of 29 nephrology patients
Bukhari *et al.* (2022)^83^	Non-invasive	Blood and ECG	48 h 12-lead ECGs, H12+, Mortara Instruments (ECG)	Not reported	Study population of 29 end stage renal disease patients

An important challenge in developing a potassium sensor for healthcare applications, as opposed to water or soil measurements, is the narrow concentration range of physiological potassium in blood and the consequences of inaccurate potassium readings, which could be potentially harmful. Blood glucose meters have improved over the last two decades to achieve an accuracy within ±20% of reference methods (*i.e.* whole numbers of mmols), but for potassium, accuracy of ±0.5 mM (ref. 84) is likely to be required for regulatory approval and clinical utility.

Additional challenges are those of haemolysis during sample collection and post-collection trans-cellular potassium movement, which are major concerns for measurement of potassium in whole blood and especially when sending blood samples to a lab for analysis. Point-of-care devices will allow healthcare professionals and patients to measure potassium on the spot without the need to transport the blood sample. However, trauma induced by a fingerprick lancet and/or finger squeezing can cause haemolysis. Sample collection technique is therefore important for potassium measurement. Although home testing would be convenient and provide more frequent and potentially less confounded monitoring, there are caveats: (a) home testing could lead to additional perceived emergency intervention or conversely, missing true hypokalemia; (b) educating both health care professionals and the public about false positive and false negatives will be essential.

The academic community is actively working on technologies for potassium detection, however, there is still a big gap between academic research and a sensor device that can be mass-produced and perform to the required accuracy. This is especially the case when trying to develop a single-use disposable test that does not require calibration before use (as with many glucometer test strips). Nevertheless, commercialization efforts are ongoing by several companies that aim to develop self-tests for home use. None has yet received regulatory approval for such a device, but this should be possible within the next few years.

Modern dialysis treatments aim to personally tailor dialysis delivery and to this end, an *in situ* means of measuring potassium content of the dialysate in real time as well as in the blood circuit would be of benefit. Current advances in haemodialysis machines, which are becoming smaller and more personalized, and measures focused on improving health outcomes and reducing treatment costs such as the Advancing American Kidney Care Initiative,^85^ will likely increase the proportion of kidney patients receiving home dialysis. With that, the need for potassium monitoring at home to safely tailor dialysis frequency and duration will increase. Lastly, ongoing efforts by researchers and companies that develop minimally painful and minimally invasive blood extraction devices will also advance the home and self-monitoring market.

As interest in personalised health monitoring and patient self-management continues to increase, so does demand for new tests. Moreover, new and exciting technologies may be developed in the future – for example, wearable biosensors could be self-powered when paired with an energy-harvesting technology to convert biomechanical, biochemical, or converting solar energy into electricity which could be used for active sensing of physiological parameters.^86^

For devices that are aimed for continuous monitoring, either on the skin, in the interstitial fluid or completely implantable, the challenges associated with materials breathability, biocompatibility and biofouling need to be addressed.^87^ This is especially important as the functionality of the sensor can change due to accumulation of sweat, non-specific cell/protein adsorption or even due leaching out of the sensor materials to the surrounding medium/tissue. Those challenges of skin-wearable and implantable biosensors, as well as efforts to improve comfortability, are an active current area of research.^88–90^

## 4. Conclusions

This review has summarized recent advances in development of potassium sensing devices for healthcare applications. We have discussed point-of-care devices for measurement of potassium in venous blood, devices for home blood collection and remote measurement, devices for rapid home measurement of potassium, potassium-sensing technologies for urine, wearable sensors for potassium in interstitial fluid, wearable sensors for potassium in sweat and non-invasive and indirect potassium detection.

While the underlying electrochemical and optical detection principles were established decades ago, the scientific community is developing innovative solutions to miniaturize devices and integrate them onto flexible substrates and wearables. Innovation in microneedle sensors, wearable sensors and flexible electronics open up opportunities for development of continuous and minimally invasive potassium sensors. However, clinical validation of the suitability of ‘non-blood’ samples for potassium determination will have to be established first. Moreover, there are additional challenges associated with developing continuous *in vivo* sensors such as: (i) it is important to verify that there are no biocompatibility risks and to use non-toxic sensor-materials, (ii) ensure that there is no biofouling during the measurement timeframe, and (iii) important to mitigate sensor drift over time which can be much more significant for potassium, compared to glucose for example, because potassium has a very narrow physiological concentration range.

It is therefore expected that in the near future, fingerprick potassium measurement will be the most realistic approach for cardiorenal home/self-management, provided potential issues with reliable sample collection, low-cost manufacturing and regulatory pathways are overcome. The introduction of fingerprick blood potassium testing into clinical use will demonstrate effectiveness and drive changes to clinical pathways.

Finally, additional areas to consider are those of privacy and data ownership and storage. Currently, lab-reported potassium levels ‘belong’ to health care providers, though in many settings patients can view or download the information. The ethical issues associated with patients themselves generating the data, how those data are stored and/or transmitted, and the security of that information, will require detailed protocol development. While those with diabetes have had decades of practice in self-determining insulin doses in response to fingerprick or wearable glucose monitoring, those with potassium disorders are very far from being in a similar position, so a multidisciplinary approach to self-testing and the data it generates will be essential.

## Conflicts of interest

The authors are shareholders in, and/or employees of Kalium Health Ltd.

## Supplementary Material
